# Making sure there's a "give" associated with the "take": producing and using open-source software in big pharma

**DOI:** 10.1186/1758-2946-3-S1-O3

**Published:** 2011-04-19

**Authors:** Gregory Landrum, Richard Lewis, Andrew Palmer, Nikolaus Stiefl, Anna Vulpetti

**Affiliations:** 1 Novartis Institutes for BioMedical Research, Basel, CH-4002, Switzerland; 2 Novartis Institutes for BioMedical Research, Cambridge MA, 02139, USA

## 

In contrast to bioinformatics, open-source software is not as widely used in the pharmaceutical industry for molecular modeling and cheminformatics. Typical reasons given for this include problems with code quality, stability, and long-term support for the software (somehow this is less of a concern with bioinformatics software... kind of makes one think). Recently, our group has started making heavy use of an open-source cheminformatics toolkit RDKit [1] in our production environment. Importantly, we are not just acting as consumers of open-source software -- we are active members of the open-source community and have support from management to contribute code back to the project.

In this presentation we will provide a brief overview of the RDKit itself and then present a number of case studies of how we have made use of this open-source platform. Examples will include using the toolkit for method development [2,3], integration with proprietary tools, and some recent (and upcoming) contributions to the open-souce community, including a database cartridge for fast and flexible similarity searching in the open-source PostgreSQL database [4], and adding support for the RDKit within the open-source pipelining platform Knime [5]. We will finish with a discussion of some practical aspects of working on and with open-source tools in a large research organization.
